# Work ability and associated factors of Brazilian technical-administrative workers in education

**DOI:** 10.1186/s13104-015-1837-x

**Published:** 2016-01-02

**Authors:** Marluce Rodrigues Godinho, Rosangela Maria Greco, Maria Teresa Bustamante Teixeira, Liliane Reis Teixeira, Maximiliano Ribeiro Guerra, Alfredo Chaoubah

**Affiliations:** Sergio Arouca National School of Public Health, Oswaldo Cruz Foundation, Rio de Janeiro, Brazil; Basic Nursing Department, School of Nursing, Federal University of Juiz de Fora, Juiz de Fora, Brazil; Department of Collective Health, School of Medicine, Federal University of Juiz de Fora, Juiz de Fora, Brazil; Center for Workers Health and Human Ecology Studies, Sergio Arouca National School of Public Health, Oswaldo Cruz Foundation, Rio de Janeiro, Brazil; Statistics Department, Federal University of Juiz de Fora, Juiz de Fora, Brazil

**Keywords:** Workers health, Assessing work ability, Population ageing, Labor conditions

## Abstract

**Background:**

Studies about work ability have grown in importance owing to the worldwide aging of active populations. Research has shown that measuring work ability has a predictive value in cases of long-term sickness absence and early retirement. Our goal was to analyze the work ability and associated factors of civil servants from a higher education institution in Brazil. The participants in this cross-sectional study were 600 technical-administrative workers at a public university. Work ability was measured using the work ability index.

**Results:**

The participants were as follows: 51.8 % male; mean age of 45 years (SD = ±11); married or in a stable union (61.5 %); holding a graduate degree (56.7 %); having only one job (83.3 %), working 40 h a week or less (78.6 %); not working evenings (79.8 %); and having direct contact with the public (58.3 %). The prevalence of reduced work ability was 13.9 %. The following factors were found to be associated with reduced work ability: age 50 years old or above (PR = 2.58; 95 % CI 1.25–5.09); female (PR = 2.77; 95 % CI 1.25–3.60); education up to secondary school (PR = 2.37; 95 % CI 1.13–3.59); overall poor self-assessed health (PR = 2.96; 95 % CI 1.32–3.93); signs and symptoms of depression (PR = 4.86; 95 % CI 2.23–6.55); sedentariness (PR = 3.00; 95 % CI 1.38–4.68) and poor social support at work (PR = 4.01; 95 % CI 1.66–4.37).

**Conclusions:**

Most of the participants showed good work ability, but some subjects had reduced work ability. This study makes a contribution to expanding the discussion about the factors associated with work ability toward proposing actions for maintaining that ability or helping recovery in the case of diminished ability. Such actions can help reduce work absenteeism and early retirement, both of which have a social and economic impact in Brazil. Studying the determinants of work ability and recommendations to address those determinants will help efforts to improve the quality of life of individuals, both at work and personally, and promote healthy aging.

## Background

Work ability is defined as the capability of a worker in the present or near future to perform the functional demands of their work with respect to their current state of health and their physical and mental capacity. Work ability is a measure of functional aging [1]. Functional aging is the loss of a person’s work ability: it often occurs before chronological aging and is preventable [2, 3]. Promoting work ability can reduce work incapacity and early retirement [4]. Sell et al. [5] demonstrated that work ability offers a viable means of measuring an individual’s capability to remain in the labor market. A cohort study conducted using a representative sample of Denmark’s working population over a 5 year period reported how self-reported work ability was able to predict long-term cases of sickness absence and early retirement [5].

Work-related diseases, such as musculoskeletal disorders, are a major cause of incapacity in active populations, and they generate social security costs in many countries [6]. The process of falling ill often leads to incapacity, which is related to diminished work ability [4]. Based on several studies about work ability and its main determinants, the Finnish Institute of Occupational Health developed the work ability index (WAI) toward establishing the main determinants, consequences, and intervention measures related to work ability and also to support a government policy proposal about maintaining that ability [1]. Based on those studies conducted in the early 1980s, researchers from several other countries have undertaken similar investigations [1].

In Brazil, studies about work ability began to emerge in the late 1990s; thereafter, several authors have studied this issue [4]. However, according to the systematic review by Martinez et al. [4], those Brazilian studies address specific groups of workers, such as health professionals, production line workers, electricians, firefighters, administrative workers, and forensic workers [4]. A notable feature of those studies is the heterogeneity of the results, which poses difficulties in comparing results among different populations of workers [4, 7]. For example, Alcantara et al. [8] undertook a study about work ability and the effects of aging, health, and work on a population of Brazilian municipal employees. The authors concluded that work ability is the result of dynamic interactions between the individual, their experiences and habits, and the environment [8].

Studies have demonstrated that reduced work ability causes individuals to leave work, either temporary or even permanently [4]. In Brazil, the most current data of the Ministry of Social Security [9] show that in 2011, expenditure on leaves of absence and special retirements related to work environment conditions totaled US$9.1 billion. This underlines the social and economic impact on public health in Brazil; the situation will be exacerbated as a result of population aging, which has affected economically active populations worldwide [10].

The International Labor Organization [11] has estimated that by 2025, the proportion of individuals over the age of 55 years will amount to 17 % in Latin America, 21 % in Asia, 30 % in North America, and 32 % in Europe [11]. This highlights the urgent need for change in public policies and appropriate action for improvement: the accompanying social changes will have consequences in several sectors, such as the labor market, owing to aging of the workforce [3, 8, 12].

Brazil has a large number of civil servants. The “civil servant” is a public office: the individual performs a public test and, if approved, acquires stability in public services. Public servants who conduct professional activities in federal educational institutions are termed technical-administrative workers in education [13].

The work of technical-administrative workers in education is a fundamentally important part of human resource development in Brazil. However, those workers have not been subject to research with respect to occupational health—particularly regarding work ability. Based on the current situation in Brazil and the urgent need to preserve the work ability of its active population, the present study aimed to analyze the prevalence of reduced work ability and associated factors among technical-administrative workers in education at the Federal University of Juiz de Fora.

## Methods

The present study is part of a wider research effort that is being conducted to investigate factors related to labor conditions, health, and living conditions of technical-administrative workers in education at the Federal University of Juiz de Fora in the city of Juiz de Fora, state of Minas Gerais, Brazil. The aim of the wider research effort is to form a cohort and follow up those workers, with updated information every 5 years. In the present study, we adopted a cross-sectional design. The inclusion criteria were as follows: being a technical-administrative worker in the field of permanent education and being active in exercising that function. The exclusion criteria were as follows: being on medical leave; being licensed to train or being a qualified professional; and working at another institution.

The period of data collection of this study was February 2012 to August 2013. Among a population of 1265 technical-administrative workers at the university, we approached 696 workers that were present at the workplace. Of those 696 technical-administrative workers (convenience sample), 31 were on medical leave of absence, five were licensed to train or were qualified professionals, and two were working at another institution. We thus considered 658 subjects to be eligible. Of those, 91.2 % (n = 600) answered the survey.

The dependent variable of work ability was obtained through answers given by the workers to the WAI survey, which was developed by researchers in Finland [1]. It has been translated and adapted to Brazil by researchers from Universities in the state of São Paulo [1]. Afterwards, it was validated by a study realized with workers from an electrical company in the state of São Paulo, Brazil [14] as a survey with closed-ended questions to be answered by the workers themselves. The instrument consists of seven items, each with one or more questions, and an individual’s score ranges from 7 to 49. The score depends on the answers to several questions about the physical and intellectual demands of work, the employees’ health status, and their resources [1]. Based on the score, work ability is classified as follows: poor (score of 7–27, recommendation to restore work ability); moderate (score of 28–36, recommendation to improve work ability); good (score of 37–43, recommendation to support work ability); and excellent (score of 44–49, recommendation to maintain work ability) [1]. As proposed by Raffone and Hennington [15], the dependent variable was categorized as reduced work ability (7–36) and good work ability (37–49) for comparison with the other study variables.

The independent variables (sociodemographic, occupational, and health conditions and life habits) were chosen in accordance with previous studies [2, 5, 7, 11]; they were based on questions about the following: age; color or race; marital status; sex; education; family income; weekly working hours; evening jobs; contact with the public; job demands; self-perceived overall health status; dental health status; and smoking status. The variables of sex and age are also important control variables. The independent variables were measured using items from the following instruments: the Patient Health Questionnaire, which we used to evaluate signs and symptoms of depression [16]; the International Physical Activity Questionnaire (short), which we employed to measure the level of physical activity [17]; the alcohol use disorders identification test, which we utilized to identify the individual’s alcohol consumption pattern [18]; the Social Support Survey used in the Medical Outcomes Study, which we used to measure social support [19]; and the Swedish Scale of social demand-control-support. We employed the demand–control model to analyze the stressors of work based on work demands and employee control over those demands. That model was elaborated by Karasek and Theorell [20, 21]. Social support in the workplace was added to the model by Johnson and Hall in 1988 [22] as a means of assessing interaction between employees and managers toward work achievement [23]. All the above instruments have been tested and validated for Brazil by Matsudo et al. [17], Lima et al. [18], Griep et al. [19], and Alves et al. [23].

Data entry and univariate analysis were performed by means of SPSS programme (version 15.0) and other analyses employed Stata programme (version 11.0). Our analysis adopted the following steps: (1) descriptive, through absolute and percentage frequencies of all the study variables; (2) bivariate analysis, in which we could identify associations between independent variables and work ability by crossing work ability with each of the independent variables through contingency tables and Chi square tests and also by verifying the gross prevalence ratio; variables that were significantly related with the WAI, at* p* ≤ 0.05, were added to the Poisson regression; (3) Poisson regression to determine which variables were most important in explaining reduced work ability by determining the adjusted prevalence ratio, 95 % confidence intervals, and* p* values.* p* values equal to or below 0.05 were considered statistically significant.

It was expected that the identified associations would promote the discussion about factors associated with work ability and that the results would support or refute the associations established in previous studies with other professional categories. We believed that our findings would allow us to recommend actions to reduce, control, or prevent diminishing work ability as well as actions to improve it.

This project was approved by the Research Ethics Committee of the Federal University of Juiz de Fora under registration number 224/2010. All participants read and signed an informed voluntary consent form after being apprised of the goals of the research and its confidential, voluntary nature.

## Results

In this study, there was a proportional distribution of gender, with 51.8 % of the subjects being men. The age ranged from 21 to 68 years, with an average of 45.1 (SD ± 11.0); the predominant age was 50 years or older (45.0 %). Most of the respondents were married or in a civil union (62.2 %) and were classified as white (68.1 %). Over half of the participants (57.2 %) had a graduate degree, and the family income of 49.1 % of the respondents ranged from 5 to 10 minimum wages.^1^

With respect to their overall health and dental status, most respondents answered “good” for both (52.2 and 51.4 %). Most subjects did not show any signs or symptoms of depression (89.5 %); 33.3 % of participants classified themselves as physically active, i.e., they performed some form of vigorous physical activity at least three times a week. With respect to smoking habits, most did not smoke or had never smoked (64.1 %). According to the performed classification for alcohol, the predominant categories were abstinence or risk-free consumption (81.5 %). Regarding social networks and aspects of their lives with family, friends, and group activities, most participants (83.7 %) stated that they had one or more relatives, and 77.5 % declared that they had one or more friends with whom they felt comfortable talking about everything or almost everything. Therefore, most of those individuals (85.1 %) had a high level of social support. With respect to group activities, most subjects said that they attended religious services or activities related to religion. By contrast, over the previous year, 68.5 % of participants stated that they had not taken part in activities related to sports or culture; 54.3 % had not attended any meetings held by community or employee associations, unions, or political parties; and 78.5 % had not taken part in any unpaid volunteer work activities.

Regarding occupational variables, most subjects (83.9 %) had only one job, worked 40 h per week or less (78.6 %), and did not work in the evenings or engage in shift work (80.9 %). Most workers had direct contact with the public (58.9 %) and their work requirements were predominantly intellectual (66.4 %). With regard to demand, control, and social support at work, the scores for each dimension were obtained by summing the points for each answer and dividing them into two categories with respect to the median, as suggested by Alves et al. [23]. The cutoff point for demand was 14 points; for control and social support at work, it was 17 points. The results indicate that 73.6 % of the participants reported low demand and 56.1 % low control. Combining demand and control at work indicated that 41.6 % of subjects had a passive job status (low demand, low control), 31.9 % had low strain job status (low demand, high control), 14.5 % had high strain job status (high demand, low control), and 12 % had active job status (high demand, high control). In all, 76.3 % of the technical-administrative workers reported strong social support at work.

The average WAI among the participants was 41.6 (SD ± 5.1), with scores ranging from 22 to 49; the prevalence of reduced work ability was 13.9 %. Non-white women aged 50 years or older and who were educated up to secondary school showed a greater prevalence of reduced work ability (Table 1).

**Table 1:** Bivariate analysis—prevalence of reduced WAI and prevalence ratio according to sociodemographic characteristics of technical-administrative workers in education at the Federal University of Juiz de Fora, Juiz de Fora, 2013 (N = 600)

Variables	Total		Reduced WAI		PR	95 % CI	p value
	N	(%)	N	(p/ %)			
Age							
Up to 34 years old	142	(24.7)	12	(8.5)	1		
35–49 years old	174	(30.3)	20	(11.5)	1.36	0.66–2.78	0.400
50 or older	258	(45.0)	44	(17.1)	2.02	1.07–3.82	0.031
Color or race (according to IBGE)							
White	399	(68.2)	46	(11.5)	1		
Non-white	186	(31.8)	35	(18.8)	1.63	1.05–2.53	0.029
Marital status							
Married	365	(62.2)	55	(15.1)	1		
Not married	222	(37.8)	27	(12.2)	0.81	0.51–1.28	0.362
Sex							
Male	305	(51.8)	27	(8.9)	1		
Female	284	(48.2)	55	(19.4)	2.19	1.38–3.47	0.001
Level of education							
University degree or higher	466	(79.5)	52	(11.2)	1		
Up to secondary school	120	(20.5)	30	(25.0)	2.24	1.43–3.51	0.000
Monthly family income							
Over 10 minimum wages	123	(21.4)	15	(12.2)	1		
5 to ≤ 10 minimum wages	280	(48.9)	45	(16.1)	1.32	0.73–2.36	0.355
≤5 minimum wages	170	(29.7)	20	(11.8)	0.96	0.49–1.88	0.916

Differences in totals are explained by the fact that some information regarding a few variables was lost. Data below 6 % not informed

*WAI* work ability index, *PR* prevalence ratio

95 % CI: 95 % confidence interval

Source: The author, 2013

Bivariate analysis indicated that there was a direct association between reduced work ability and poor self-assessment of general health and dental status, signs and symptoms of depression, sedentariness, and alcohol addiction. Low social support, not having a relative with whom the subject felt comfortable confiding in, and not taking part in sports activities were all directly associated with a decline in the work ability of the participants (Table 2).

**Table 2:** Bivariate analysis—prevalence of reduced WAI and prevalence ratio according to health conditions and life habits of technical-administrative workers in education at the Federal University of Juiz de Fora, Juiz de Fora, 2013 (N = 600)

Variables	Total		Reduced WAI		PR	95 % CI	p value
	N	(%)	N	(p/ %)			
Self-assessed overall health status							
Good	516	(87.5)	54	(10.5)	1		
Bad	74	(12.5)	28	(37.8)	3.61	2.29–5.71	0.000
Self-assessed overall dental status							
Good	436	(75.0)	53	(12.2)	1		
Bad	145	(25.0)	28	(19.3)	1.59	1.00–2.51	0.048
Signs and symptoms of depression							
Absent	508	(89.8)	48	(9.4)	1		
Present	58	(10.2)	28	(48.3)	5.11	3.21–8.14	0.000
Level of physical activity							
Active or very active	320	(54.8)	37	(11.6)	1		
Insufficiently active	190	(32.5)	25	(13.2)	1.14	0.69–1.89	0.618
Sedentary	74	(12.7)	19	(25.7)	2.22	1.23–3.86	0.005
Alcohol addiction							
Risk-free consumption	472	(81.2)	62	(13.1)	1		
Risky consumption	98	(16.9)	14	(14.3)	1.09	0.61–1.94	0.777
Addiction	11	(1.9)	5	(6.2)	3.46	1.39–8.61	0.008
Smoking							
Non smoker	513	(88.9)	68	(13.3)	1		
Smoker	64	(11.1)	11	(17.2)	0.77	0.41–1.46	0.424
Social network (relatives)							
One or more relatives	495	(83.9)	56	(11.3)	1		
None	95	(16.1)	26	(27.4)	2.42	1.52–3.85	0.000
Social network (friends)							
One or more friends	460	(78.0)	57	(12.4)	1		
None	130	(22.0)	25	(19.2)	1.55	0.97–2.48	0.067
Social network (sports activities)							
Yes	185	(31.6)	17	(9.2)	1		
No	400	(68.4)	63	(15.8)	1.71	1.00–2.93	0.032
Social network (meetings)							
Yes	264	(45.4)	30	(11.4)	1		
No	317	(54.6)	50	(15.8)	1.39	0.88–2.18	0.156
Social network (volunteer work)							
Yes	127	(21.8)	15	(11.8)	1		
No	456	(78.2)	66	(14.5)	1.23	0.70–2.15	0.477
Social network (religious activities)							
Yes	478	(81.3)	69	(14.4)	1		
No	110	(18.7)	12	(10.9)	0.76	0.41–1.39	0.371
Social support							
Strong support	480	(85.0)	53	(11.0)	1		
Low support	85	(15.0)	22	(25.9)	2.34	1.43–3.85	0.001

Differences in n totals are explained by the fact that some information regarding a few variables was lost. Data below 6 % not informed

*WAI* work ability index, *PR* prevalence ratio

95 % CI: 95 % confidence interval

Source: The author, 2013

Using the social demand–control–support model, we found an association between reduced work ability and stressors in the workplace and low social support at work. We also determined that contact with the public and the type of work function were associated with reduced work ability (Table 3).

**Table 3:** Bivariate analysis—prevalence of reduced WAI and prevalence ratio according to work characteristics of Technical-Administrative Workers in Education at the Federal University of Juiz de Fora, Juiz de Fora, 2013 (N = 600)

Variables	Total		Reduced WAI		PR	95 % CI	p value
	N	(%)	N	(p/ %)			
Number of jobs							
One	492	(83.8)	68	(13.8)	1		
Two or more jobs	95	(16.2)	14	(14.7)	1.06	0.60–1.90	0.827
Weekly working hours							
≤40 h	453	(78.5)	62	(13.7)	1		
>40 h	124	(21.5)	16	(12.9)	0.94	0.54–1.63	0.834
Evening jobs							
No	472	(80.7)	62	(13.1)	1		
Yes	113	(19.3)	18	(15.9)	0.98	0.92–1.04	0.471
Social support at work							
Strong social support	443	(76.5)	43	(9.7)	1		
Low social support	136	(23.5)	38	(27.9)	2.88	1.86–4.45	0.000
Demand-control model^a^							
Low strain job	183	(32.1)	18	(9.8)	1		
Active job	69	(12.1)	5	(7.2)	0.74	0.27–1.98	0.546
Passive job	236	(41.3)	34	(14.4)	1.46	0.83–2.59	0.190
High strain job	83	(14.5)	24	(28.9)	2.94	1.60–5.42	0.001
Contact with the public							
Indirect or no contact	109	(18.6)	7	(6.4)	1		
Direct or direct and indirect	478	(81.4)	75	(15.7)	2.44	1.13–5.30	0.024
Type of work function							
Mixed	136	(23.1)	35	(25.7)	1		
Physical	62	(10.5)	10	(16.1)	0.63	0.31–1.26	0.193
Mental	392	(66.4)	37	(9.4)	0.37	0.23–0.58	0.000

Differences in n totals are explained by the fact that some information regarding a few variables was lost. Data below 6 % not informed

*WAI* work ability index, *PR* prevalence ratio

95 % CI: 95 % confidence interval

^a^ Karasek, 1979

Source: The author, 2013

The final multivariable model (Poisson regression) included sociodemographic and occupational variables, health conditions, and life habits: in the bivariate analysis, those factors presented a significant association with reduced work ability (*p* ≤ 0.05). The variables included in the Poisson regression were as follows: age; color or race; sex; level of education; self-assessed overall health status; self-assessed overall dental status; signs and symptoms of depression; level of physical activity; alcohol addiction; social network (relatives); social network (sports activities); social support; social support at work; demand–control model; contact with the public; and type of work function.

After the Poisson regression, the variables that remained significantly associated with reduced work ability were age, sex, level of education, self-assessed overall health status, signs and symptoms of depression, level of physical activity, and social support at work (Table 4).

**Table 4:** Poisson regression—Gross and adjusted prevalence ratio according to factors associated with reduced WAI of Technical-Administrative Workers in Education at the Federal University of Juiz de Fora, Juiz de Fora, 2013 (N = 538)

Variables	PR Gross	95 % CI	p value	PR Adjusted	95 % CI	p value
Age						
Up to 34 years old	1			1		
35 to 49 years old	1.36	0.66–2.78	0.400	1.33	0.79–3.42	0.182
50 or older	2.02	1.07–3.82	0.031	2.58	1.25–5.09	0.010
Sex						
Male	1			1		
Female	2.19	1.38–3.47	0.001	2.77	1.25–3.60	0.006
Level of education						
University degree or higher	1			1		
Up to secondary school	2.24	1.43–3.51	0.000	2.37	1.13–3.59	0.018
Self-assessed overall health						
Good	1			1		
Bad	3.61	2.29–5.71	0.000	2.96	1.32–3.93	0.003
Signs and symptoms of depression (PHQ9)						
Absent	1			1		
Present	5.11	3.21–8.14	0.000	4.86	2.23–6.55	0.000
Level of physical activity						
Active or very active	1			1		
Insufficiently active	1.14	0.69–1.89	0.618	0.01	0.57–1.75	0.995
Sedentary	2.22	1.23–3.86	0.005	3.00	1.38–4.68	0.003
Social support at work						
Strong social support	1			1		
Low social support	2.88	1.86–4.45	0.000	4.01	1.66–4.37	0.000

*WAI* work ability index, *PR* prevalence ratio

95 % CI: 95 % confidence interval

Source: The author, 2013

Female subjects evidenced a greater probability of reduced work ability. There was a significant association between reduced work ability and age of 50 years or above. We found a direct association between lower levels of education and reduced work ability. Reduced work ability was likewise associated with a poor self-assessment of general health and signs and symptoms of depression. Sedentariness was directly and significantly associated with reduced work ability. Only one occupational variable showed a significant association after the Poisson regression: there was a direct association between social support at work and work ability.

## Discussion

We found the prevalence of reduced work ability among technical-administrative workers in education at Federal University of Juiz de Fora to be 13.9 %. This proportion is similar to the reduced work ability of 11.3 % observed by Fischer and Martinez [24] among nursing professionals at a hospital in São Paulo, Brazil. By contrast, the percentage of workers with reduced work ability determined in the present study is lower than that reported in other Brazilian studies: 20.4 % for workers in hospital housekeeping services [25]; 34.5 % for workers in sawmills [26]; 35.3 and 46 % for municipal school teachers [27, 28]; and 43.3 % in nurses [29].

The differences may be explained by the different sociodemographic and occupational profile of the present study population compared with that of other groups of Brazilian workers, especially regarding the type of employment contract. The workers who took part in the present study had job stability; that is contrast to workers in unstable, outsourced employment, which has grown in all industries in Brazil in recent years and has had an impact on the working conditions of the entire population [30].

In the present study, reduced work ability was found to be associated with individual characteristics, health conditions, life habits, and work-related factors: being of an older age; being female; having a low level of education; having a poor self-assessed overall health status; having signs and symptoms of depression; having a low level of physical activity; and having low social support at work. This result is similar to that obtained by Fischer and Martinez [24], who found that reduced work ability was associated with sex, sedentariness and social support at work. The result is also similar to that obtained by Van den Berg et al. [7], who identified the factors associated with poor work ability to be older age, lack of leisure time, lack of vigorous physical activity, poor musculoskeletal capacity, obesity, high physical and psychosocial work demands, poor physical work environment, and high physical workload [7, 24].

We found that such factors as color, self-assessed overall dental status, alcohol addiction, network and social support, work stressors, contact with the public, and type of work function did not continue to show a significant association after the Poisson regression. That is possibly because other variables were more strongly associated with work ability.

In the present study, age was observed to be one of the factors significantly associated with work ability. Older workers aged 50 years or above displayed a greater probability of reduced work ability. This finding is in accordance with that of Raffone and Hennington [15], who reported the prevalence of reduced work ability among nursing professionals to be 16.8 %. Those authors also observed diminishing work ability with advancing age. However, having a preexisting condition may exert a greater negative effect on an individual’s work ability than age [31]. Marqueze and Moreno [32] studied college educators at a university in Santa Catarina, Brazil and found the prevalence of reduced work ability to be 13 %, which is very similar to our own result. However, in contrast to the present study, those authors maintained that functional aging was not necessarily related with chronological aging: they believed that functional aging depends on individual characteristics and on adopted lifestyles in addition to living and working conditions [32].

We found sex to be associated with reduced work ability: women showed a greater likelihood of reduced work ability. One possible explanation for this is the greater burden for women. Although women have increasingly become a part of the labor market in Brazil and achieved independence and equal rights with men in several respects, home chores and taking care of their children and husbands is frequent still a woman’s responsibility. Domestic demands can impose a double or even triple burden on women. Female employees have roles that extend beyond those of work, such as those of mother and wife, which have an impact on their health and work ability [32, 33].

In the present study, the level of education was significantly associated with the outcomes: developing actions related to health education and encouraging people to pursue healthier lifestyles is easier with individuals having higher levels of education, and that contributes to improved work ability [34]. We found that the higher education level of the technical-administrative workers—over half of them had a graduate degree—could explain the prevalence of good work ability.

Life habits and health conditions were also found to be significantly associated with work ability. The significant association between work ability and health could be owing to the conceptual overlap between them: question 4 of the WAI includes statements related to diseases [1]. Studies have shown that health conditions are crucial with respect to quality of life and work ability [35], and self-assessed health is one of the most widely used public health indicators [36]. In a study of teachers in São Paulo conducted by Vedovato and Monteiro [27], a poor self-assessment of general health was found to be associated with reduced work ability. In their study, most of the respondents were women, with a mean age of 41.4 years and a high level of education; the prevalence of reduced work ability was 35.4 %. It should be noted that the results of the present study are in accordance with those of Silva Junior et al. [37], who found a direct, significant correlation between the WAI and self-assessed health status. The authors observed that employees with greater work ability were usually those who had a more positive outlook on their overall health status [37].

We found that sedentariness and signs and symptoms of depression were directly associated with a decline in work ability [15]. In 2001, severe depression was the main cause of decreased work ability, and it ranked fourth among the top ten causes of illness worldwide [38]. According to a recent forecast by the World Health Organization [39], depression will be the most prevalent worldwide illness by 2030. Therefore, sedentary individuals and those with depressive symptoms have a greater chance of becoming incapacitated for work than those who exercise regularly and lack such symptoms [40].

Among occupational variables, only one retained a significant association after the Poisson regression: social support at work was related to the interaction between employees and managers in job cooperation [23]. We found low social support at work to be associated with poor work ability, and it could contribute to increased worker weakness and health risks [23]. Improved work ability is strongly associated with better relationships with supervisors and in the organizational process of work [41]. Negeliskii and Lautert [42] consider social support to be the foundation of labor relationships and a social organization strategy in institutions. Social support would thus appear to be a way to reduce and even prevent work stress. By valuing relationships and the work environment, it is possible to promote the benefits to employees’ health and their work ability, which could contribute to reduce absenteeism caused by illness and early retirement [42].

It is necessary to note that the present study could be subject to a healthy worker bias: incapacitated workers miss work more often, are on sick leave for treatment, or have retired. It should also be noted that this study’s cross-sectional design provides a single snapshot of the relationships under scrutiny. Such cross-sectional cohort studies can lead to the identification of just the survivors of the effect under study (prevalence bias), and this could lead to underestimating the level of risk in the assessed work process [43].

Furthermore, it is not possible with cross-sectional studies to establish the directions of causal relationships. It is likewise not possible to determine health aspects before the study period or forecast how those aspects will develop in the future. There is a possibility with the present study that low work ability may have stimulated certain life habits and healthy behaviors, such as exercising, though exercising did not have an impact on an individual’s work ability.

Other limitations that deserve mention are the type of questionnaire used. The self-completed questionnaire depends on an individual’s report and can be influenced by such factors as memory, understanding capacity, and the bias of socially desirable reporting. El Fassi et al. [44] have argued that workers who perceive their health to be declining could decide not to complete a questionnaire or to provide incorrect details through fear of influencing the occupational physician’s decision concerning their fitness for work.

Although this study has the above limitations, it has the benefit of expanding the discussion about the factors associated with reduced work ability in a working population. This discussion can form the basis for developing actions and policies to support employees’ work ability and help the recovery of those with inadequate ability.

## Conclusions

The results of this study indicate that developing healthy life habits and reorganizing the work environment should be encouraged by stimulating employees to undertake physical activity, have healthy eating habits, and reinforce knowledge of the harmful effects that such habits as smoking and alcoholism can exert on health. Reorganization of the work environment should allow interaction between employees and managers toward improving personal relationships in the workplace. This could lead to better work ability and perhaps prevent early retirement, which has a social and economic impact in Brazil. However, more important than reducing the social and economic impact is preventing worker illness and improving their quality of work life.

Despite this study’s limitations, it is necessary to emphasize the importance of assessing work ability in this population because 13.9 % of the employees had reduced work ability. These data show the need for urgent local intervention to prevent permanent illness and early retirement of these workers.

It is necessary, however, to conduct longitudinal studies to clarify the directions of the identified associations and to analyze possible changes. Such studies could lead to the development of effective interventions to prevent factors that are harmful to health and could lead to work inability and absenteeism. The present study is part of a greater research effort, and we emphasize the need for this population to be followed up. We are already conducting a longitudinal study, and we estimate that our results will be available for publication in 3 years.

## Footnotes

1 In Brazil, there is a minimum value determined by the Government, which must be paid in cash to workers by employers and to this minimum value is given the minimum wage name.
